# DRG-LLaMA : tuning LLaMA model to predict diagnosis-related group for hospitalized patients

**DOI:** 10.1038/s41746-023-00989-3

**Published:** 2024-01-22

**Authors:** Hanyin Wang, Chufan Gao, Christopher Dantona, Bryan Hull, Jimeng Sun

**Affiliations:** 1 Division of Hospital Internal Medicine, Mayo Clinic Health System, Mankato, MN USA; 2 Department of Computer Science, University of Illinois Urbana-Champaign, Champaign, IL USA; 3 Enterprise Inpatient Clinical Documentation Integrity, Mayo Clinic, Rochester, MN USA; 4 Division of Hospital Internal Medicine, Mayo Clinic, Phoenix, AZ USA; 5 Carle Illinois College of Medicine, University of Illinois Urbana-Champaign, Champaign, IL USA

**Keywords:** Data processing, Computational models

## Abstract

In the U.S. inpatient payment system, the Diagnosis-Related Group (DRG) is pivotal, but its assignment process is inefficient. The study introduces DRG-LLaMA, an advanced large language model (LLM) fine-tuned on clinical notes to enhance DRGs assignment. Utilizing LLaMA as the foundational model and optimizing it through Low-Rank Adaptation (LoRA) on 236,192 MIMIC-IV discharge summaries, our DRG-LLaMA -7B model exhibited a noteworthy macro-averaged F1 score of 0.327, a top-1 prediction accuracy of 52.0%, and a macro-averaged Area Under the Curve (AUC) of 0.986, with a maximum input token length of 512. This model surpassed the performance of prior leading models in DRG prediction, showing a relative improvement of 40.3% and 35.7% in macro-averaged F1 score compared to ClinicalBERT and CAML, respectively. Applied to base DRG and complication or comorbidity (CC)/major complication or comorbidity (MCC) prediction, DRG-LLaMA achieved a top-1 prediction accuracy of 67.8% and 67.5%, respectively. Additionally, our findings indicate that DRG-LLaMA ’s performance correlates with increased model parameters and input context lengths.

## Introduction

The emergence of LLMs, such as GPT-3^1^ and InstructGPT^2^, has brought about a transformative shift in the landscape of Natural Language Processing (NLP). These LLMs have demonstrated exceptional capabilities across many NLP tasks in the general domain. However, the integration of LLMs into the medical field remains at a nascent stage within the academic community. Recent instances of progress highlight their significant potential, including OpenAI’s GPT-4^3^, Google’s Med-PaLM2^4^, and Google Deepmind’s Med-PaLM M^5^. GPT-4 and Med-PaLM 2 have achieved impressive performance on the United States Medical Licensing Examination (USMLE), and Med-PaLM M can even classify radiology images. Nonetheless, the medical domain introduces elevated concerns regarding safety and privacy, necessitating detailed analysis regarding the performance and limitations of LLMs to address the inherent risks such as hallucination, bias, and reasoning deficiencies^6^.

Since its inception by Medicare in 1983, DRG has served as the foundation for the inpatient prospective payment system within the United States^7^. Each distinct DRG code is delineated by a particular set of patient attributes, including principal diagnosis, specific secondary diagnoses, procedures, sex and discharge status^8^. Traditionally, the assignment of DRGs constitutes a labor-intensive manual endeavor undertaken by coding specialists, typically subsequent to a patient’s discharge. Given the pivotal role of DRGs and their bundled metrics (e.g., case-mix index, geometric length of stay) in the operational and financial performance of hospitals, a pressing interest exists in the accurate early prediction of DRGs during a patient’s hospitalization. This prediction is vital for efficacious resource planning and allocation. The task of DRG prediction presents distinct challenges compared to automated International Classification of Diseases (ICD) coding. This distinction stems from differences in the nature of the task: DRGs involve multi-class classification, where one DRG code is assigned to each visit, in contrast to the multi-label classification of ICDs, where multiple codes may apply to a single visit^9^. Additionally, the hierarchical structure of the codes, such as the presence of a principal diagnosis in DRGs, and the context of utilization in hospital operations further differentiate the two tasks^8^. Previous studies have showcased advancements in DRGs classification accuracy through various machine-learning algorithms^10^ and deep neural networks^11^. More recently, a deep learning-based NLP model leveraging adjusted Convolutional Attention for Multi-Label Classification (CAML) has been applied to predict DRGs based on clinical notes and yielded promising outcomes^12,13^.

With LLM’s remarkable natural language synthesis and generating capabilities, we hypothesize LLM could be applied to effectively predict DRGs directly from clinical notes. In this work, we present DRG-LLaMA, a fine-tuned LLM derived from LLaMA^14^. DRG-LLaMA is trained on discharge summaries from the MIMIC-IV dataset for the task of DRG prediction. In our investigation, we approached DRG prediction from two perspectives: 1) as a single-label classification task, where the model makes an end-to-end prediction of the DRG label, and 2) as a two-label classification task, where the model predicts base DRG and CC/MCC status as two separate labels, followed by the inference of the final DRG label from these two components (i.e., base DRG and CC/MCC status). Our work revealed superior performance of DRG-LLaMA in DRG prediction compared to the previously reported leading models of CAML^13^ and ClinicalBERT^15^.

## Results

### Study cohort

**Fig. 1:**
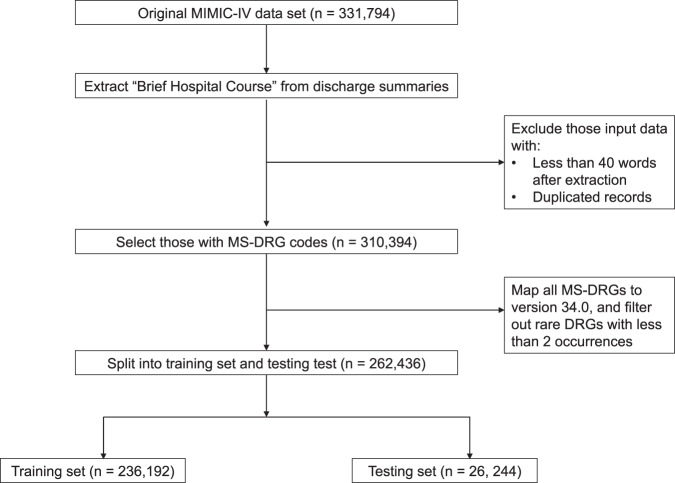
Flow diagram of the cohort processing steps. We used regular expressions to extract the “brief hospital course” section from discharge summaries in MIMIC-IV dataset as input text. We filtered the discharge summaries that were of low quality, identified by either duplicated content or containing less than 40 words. We focused on MS-DRGs and consolidated all MS-DRG codes to version 34.0. Additionally, we filtered out rare DRGs with less than 2 occurrences in the cohort.

A summary of the study cohort and data preprocessing steps was shown in Fig. 1. We focused on hospital stays with Medicare severity-DRGs (MS-DRGs) within the MIMIC-IV dataset. The “brief hospital course” section from discharge summary was extracted to serve as input text. We also filtered out low-quality discharge summaries and rare DRGs with less than 2 occurrences in the cohort. 90% of the data was allocated as training set while the rest 10% as testing set, and this partitioning was stratified on DRGs. The training and testing set contains 738 and 723 unique DRG labels, respectively. There is no significant difference in the average word counts in the training vs. testing set (398 vs. 399; *p* = 0.51 from two-sided t-test). The distribution of cases per DRG is imbalanced, with a median number of 124.5 in the training set (Supplementary Fig. 1).

### DRG prediction as a single-label classification task

**Fig. 2:**
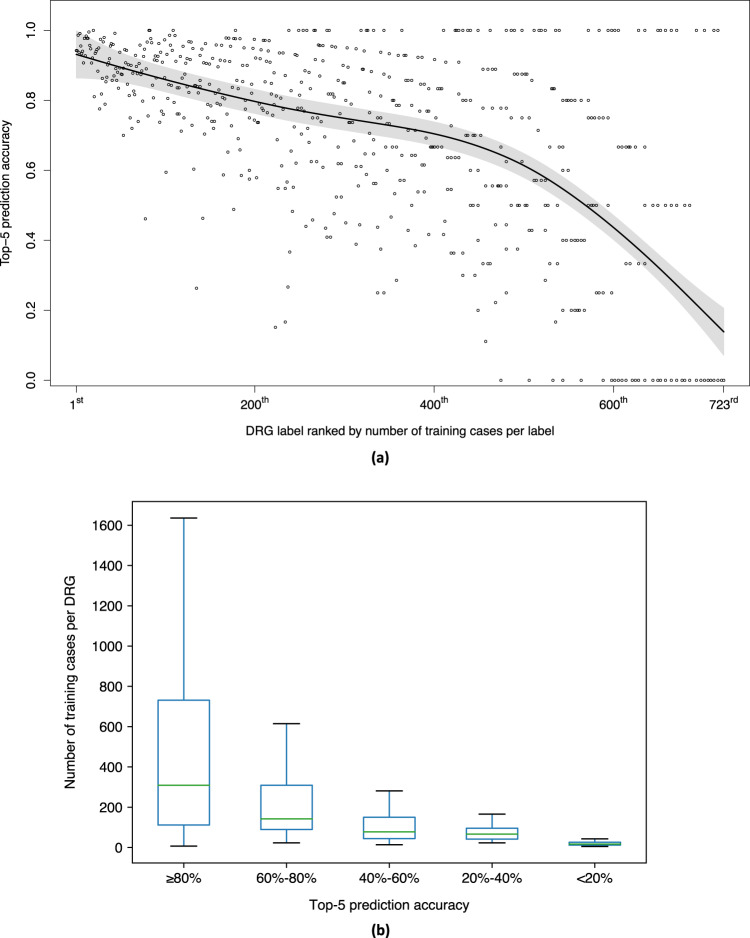
Relationship between training cases per DRG and prediction accuracy by DRG-LLaMA. Results from DRG-LLaMA -7B with a maximum input token size of 512. **a** Scatter plot of top-5 prediction accuracy versus DRG ranks by number of training cases. Y-axis is top-5 prediction accuracy of each DRG label. X-axis is the rank of the 723 DRGs by their number of training cases, where DRG ranked 1^st^ has the most training cases, and DRG ranked 723^rd^ has the least training cases. Black dots indicate individual DRGs. The solid line represents smoothing spline estimated relationship (generalized cross-validation score: 0.055). The gray shaded area denotes a 95% Bayesian confidence interval for the smoothing spline estimated function. As expected, DRG-LLaMA ’s performance declined in less frequent DRGs. **b** Boxplot of training cases per DRG with groups of different prediction accuracy. DRGs are grouped by range of top-5 prediction accuracy as shown in X-axis. Y-axis is the number of training cases per DRG. The green line represents the median value; the box limits show the interquartile range (IQR) from the first (Q1) to third (Q3) quartiles; the whiskers extend to the furthest data point within Q1-1.5*IQR (bottom) and Q3+1.5*IQR (top). DRG groups with better prediction performance generally have a greater number of training cases, although there is a large variance in the number of training cases within the best-performing group.

We presented the results with a maximum input token size of 512 in Table 1. DRG-LLaMA consistently outperformed ClinicalBERT and CAML across all evaluation metrics, with the most notable contrast seen in macro-F1 score (showing a relative improvement of 40.3% and 35.7% compared to ClinicalBERT and CAML, respectively). The accuracy of top-1 and top-5 predictions achieved by our fine-tuned DRG-LLaMA -7B model was 52.0% and 84.8%, respectively. When only considering the most frequent 300 DRGs, the top-1 accuracy improved to 55.7%, and this further increased to 69.4% in the most frequent 30 DRGs. As expected, DRG-LLaMA ’s performance declined in less frequent DRGs (Fig. 2a). When compared to CAML, ClinicalBERT achieved higher AUC and top-1 prediction accuracy but lower macro-averaged F1 score. High AUC scores were obtained for all models due to the many infrequent DRG classes, resulting in high true negative predictions for all negative class predictions^13^.

**Table 1:** Main Results on DRG prediction with a max input token size of 512.

Model	DRG set	MACRO-F1	ACC@1	ACC@5	ACC@10	MACRO-AUC	MICRO-AUC	Number (%) of cases
DRG-LLaMA -7B	All DRGs	**0.327 (0.004)**	**0.520 (0.003)**	**0.848 (0.002)**	**0.912 (0.002)**	**0.986 (0.001)**	**0.994 (0.000)**	26,244 (100.0)
	Top 300 DRGs	0.497 (0.005)	0.557 (0.004)	0.876 (0.002)	0.932 (0.001)	0.988 (0.000)	0.995 (0.000)	22,940 (87.4)
	Top 50 DRGs	0.700 (0.004)	0.666 (0.004)	0.931 (0.002)	0.965 (0.001)	0.989 (0.000)	0.998 (0.000)	10,270 (39.1)
	Top 30 DRGs	0.737 (0.005)	0.694 (0.005)	0.941 (0.003)	0.971 (0.002)	0.988 (0.001)	0.998 (0.000)	7,666 (29.2)
ClinicalBERT	All DRGs	0.233 (0.003)	0.502 (0.003)	0.815 (0.002)	0.881 (0.002)	0.979 (0.001)	0.991 (0.000)	26,244 (100.0)
CAML	All DRGs	0.241 (0.003)	0.447 (0.002)	0.785 (0.002)	0.865 (0.002)	0.976 (0.001)	0.991 (0.000)	26,244 (100.0)

F1 and AUC scores were calculated using macro-averaged or micro-averaged method as shown in the header. Notably, in a multi-class classification problem, micro-averaged F1 score is equal to top-1 prediction accuracy when labels of all classes are considered. Accuracy @1, @5 and @10 measure whether the top-1, top-5 and top-10 predictions by the model contain correct DRG code, respectively. Standard deviations are shown in parentheses and calculated using a bootstrapping procedure. Top DRGs are selected based on the number of cases per DRG in the dataset. Number (%) of cases represents hospital stays covered by the given DRG group in the testing set. Bolded scores denote the best performance with respect to the task. DRG-LLaMA outperformed ClinicalBERT and CAML across all evaluation metrics, with better performance in more frequent DRGs. *DRG* denotes diagnostis-related group, *AUC* denotes area under the receiver operating characteristic curve, and *ACC* denotes accuracy.

We investigated DRG-LLaMA ’s performance across varying model sizes and input context lengths (Table 2), observing a consistent improvement in all evaluation metrics with larger models and longer input contexts, measured in maximum token numbers. The optimal configuration, utilizing a 13B LLaMA model and a maximum input token size of 1024, achieved a top-1 prediction accuracy of 54.6%, a top-5 prediction accuracy of 86.5%, and a macro-F1 score of 0.361.

**Table 2:** DRG-LLaMA performance on different model and max input token sizes.

Model size	Max input token size	MACRO-F1	ACC@1	ACC@5	ACC@10	MACRO-AUC	MICRO-AUC
13B	1024	**0.361 (0.004)**	**0.546 (0.003)**	**0.865 (0.002)**	**0.925 (0.001)**	**0.986 (0.001)**	**0.994 (0.000)**
	512	0.334 (0.005)	0.524 (0.002)	0.853 (0.002)	0.914 (0.002)	0.984 (0.001)	0.993 (0.000)
	340	0.312 (0.006)	0.499 (0.003)	0.834 (0.002)	0.902 (0.002)	0.983 (0.001)	0.992 (0.000)
7B	1024	0.346 (0.004)	0.539 (0.003)	0.861 (0.002)	0.923 (0.001)	0.986 (0.001)	0.994 (0.000)
	512	0.327 (0.004)	0.520 (0.003)	0.848 (0.002)	0.912 (0.002)	0.986 (0.001)	0.994 (0.000)
	340	0.303 (0.005)	0.493 (0.003)	0.828 (0.002)	0.896 (0.002)	0.981 (0.001)	0.992 (0.001)

Experiments were performed on LLaMA with a size of 7 billion and 13 billion parameters. Bolded scores denote the best performance. We observed that DRG-LLaMA ’s performance consistently improved with larger models and longer input contexts.

### DRG prediction as a two-label classification task

In the two-label approach, we first dissect each DRG into two distinct components: a base DRG label and a CC/MCC label (denoting complication or comorbidity / major complication or comorbidity). This dissection process was based on the composition delineated within the MS-DRG v34.0 definitions manual^8^. The five distinct labels attributed to CC/MCC are as follows: “without CC/MCC”, “with CC”, “with MCC”, “without MCC”, and “not applicable”. As an example, in DRG code 53 of “spinal disorders and injuries without CC/MCC,” “spinal disorders and injuries” represents the base DRG label, while “without CC/MCC” serves as the CC/MCC label. Following this mapping process, the 738 DRG codes were converted into a combination of 340 base DRG labels each paired with one of the five CC/MCC labels. Results of the two-label approach using DRG-LLaMA -7B with a maximum input token size of 512 was shown in Table 3. The top-1 prediction accuracy for base DRG and CC/MCC reached 67.8% and 67.5%, respectively. This result suggests that predicting the principal diagnosis or procedure without considering CC/MCC is a significantly easier task on its own.

**Table 3:** Main Results on DRG prediction as a two-label task with a max input token size of 512.

Component	MACRO-F1	ACC@1	ACC@5	ACC@10	MACRO-AUC	MICRO-AUC	Number of labels
Base DRG	0.520 (0.005)	0.678 (0.002)	0.912 (0.001)	0.953 (0.001)	0.990 (0.001)	0.995 (0.000)	340
CC/MCC	0.680 (0.003)	0.675 (0.003)	–	–	0.909 (0.001)	0.918 (0.001)	5
DRG	–	0.515 (0.003)	–	–	–	–	738

Experiments were performed with DRG-LLaMA -7B and a maximum input token size of 512. The top-1 prediction accuracy for base DRG and CC/MCC reached 67.8% and 67.5%, respectively. A top-1 prediction accuracy of 51.5% was achieved by employing the mapping rule on base DRG and CC/MCC labels, as elaborated in the method section.

Upon integrating a mapping rule designed to infer DRGs through the combination of base DRG and CC/MCC labels, the accuracy reached 51.5% across all DRGs. Notably, this performance was comparable with the accuracy attained in the single-label approach of 52.0% using the same base model, showing that the LLM was able to achieve state-of-the-art performance via either classification setting.

### Error analysis

As noted above, a correlation exists between the number of training cases and prediction performance. DRGs with a top-5 prediction accuracy exceeding 80% are associated with a median of 309 training cases per label. In contrast, those DRGs with a top-5 accuracy below 20% are associated with only a median of 17 training cases per label (as shown in Fig. 2b). However, other factors, such as the type of DRG, also affect prediction performance. For instance, out of the DRGs with a top-1 prediction accuracy of 100%, 8 out of 9 are surgical DRGs, which have distinct hospital courses that make them easier for the model to comprehend (as listed in Supplementary Table 2). We randomly selected 10 samples from the subset where the model presented erroneous predictions within its top ten outcomes for manual error analysis (as listed in Table 4). Broadly, the identified errors were categorized as follows: erroneous CC/MCC (1/10), correct information needed for DRG prediction unavailable (1/10), difficulty in selecting correct base DRG (3/10), inadequate clinical concept extraction (4/10) and an isolated case of a plausible incorrect DRG label (1/10). Certain errors, like inadequate clinical concept extraction, indicate the model’s weaknesses. Other errors, such as the difficulty in selecting the base DRG, likely stem from the intricacies of the DRG assignment rules. Furthermore, errors such as the unavailability of correct information required for DRG prediction underscore the limitations of solely relying on discharge summaries for DRG predictions.

**Table 4:** Example of incorrect DRG predictions.

Case ID	Pertinent narratives in discharge summary	True DRG	Predicted DRG	Comment
Case 1	altered mental status…respiratory failure…acute blood loss anemia and anemia of chronic disease…clostridium difficile infection…hypotension…was initially on levophed and dopamine…	Heart failure and shock with mcc	Respiratory system diagnosis with ventilator support 96 hours	Difficulty in selecting base DRG
Case 2	gastrointestinal bleeding…most likely ischemic colitis…viral gastroenteritis…acute renal failure…anemia…	Renal failure with cc	Other digestive system diagnoses with cc	Difficulty in selecting base DRG
Case 3	worsening diabetic foot ulcer…diabetic foot infection…svt…cardiology was consulted…	Cellulitis without mcc	Diabetes with cc	Inadequate clinical concept extraction
Case 4	neutropenic fevers…infectious workup was negative except for a urine culture growing enterococcus…pt is neutropenic, thrombocytopenic, and anemic…hiv-stable…	Kidney and urinary tract infections without mcc	Major hematological and immunological diagnoses except sickle cell crisis and coagulation disorders with mcc	Difficulty in selecting base DRG
Case 5	reported chest pain…soliatry episode of nsvt..ua without pyuria…safe for d/c home…	Esophagitis gastroenteristis and miscellaneous digestive disorders without mcc	Cardiac arrhythmia and conduction disorders with cc	Correct information needed for DRG prediction not available
Case 6	septic arthritis, likely seeded by her recurrence of her e. coli bacteremia…rheum and id recommend wash out…wash out was deferred by orthopedics…	Septicemia or severe sepsis without mv 96 hours with mcc	Revision of hip or knee replacement with mcc	Inadequate clinical concept extraction
Case 7	acute to subacute hyponatremia…admitted with low na 120…uti with evidence of pyuria…	Kidney and urinary tract infections without mcc	Renal failure with cc	Inadequate clinical concept extraction
Case 8	presents with diffuse acute-on-chronic abdominal pain…gi bleed…treated with octreotide drip and pantoprazole iv…capsule endoscopy was performed…encephalopathy…visual hallucinations…	Septicemia or severe sepsis without mv 96 hours with mcc	G.i. hemorrhage with cc	Possible incorrect DRG label
Case 9	admitted for altered mental status…delirium….silent aspiration for which received a peg tube…hypertension treated with amlodipine…osa	Esophagitis gastroenteristis and miscellaneous digestive disorders without mcc	Esophagitis gastroenteritis and miscellaneous digestive disorders with mcc	Erroneous cc/mcc
Case 10	presents with word finding difficulties and lethargy…eeg showed moderate encephalopathy…ams was likely due to overmedication…followed by psychiatry - seroquel and abilify were held…	Psychoses	Other disorders of nervous system with cc	Inadequate clinical concept extraction

We manually reviewed 10 cases for error analysis. For each case, we extracted most pertinent medical problems and their narratives from discharge summaries. Certain errors, like inadequate clinical concept extraction, indicate the model’s weaknesses. Other errors, such as the difficulty in selecting the base DRG, likely stem from the intricacies of the DRG assignment rules. Furthermore, errors such as the unavailability of correct information required for DRG prediction underscore the limitations of solely relying on discharge summaries for DRG predictions.

## Discussion

Language models based on the transformer architecture, either pretrained or fine-tuned using biomedical corpora, have demonstrated efficacy across a spectrum of NLP benchmarks within the biomedical realm^16–18^. When contrasted with their predecessors rooted in the BERT architecture^19^, LLMs stand out due to their substantial size and their pretraining on expansive, cross-disciplinary text corpora. LLMs exhibit a notable capacity for comprehending and reasoning with clinical knowledge. Without domain-specific fine-tuning or specialized prompt crafting, GPT-4 exceeded the passing score on USMLE by over 20 points and set a new state-of-the-art^3^. On this premise, it is plausible to speculate that once attuned to the medical domain, an LLM could deliver robust performance across diverse NLP tasks, including the prediction of DRGs.

Toward deploying a local LLM, we used LLaMA, a robust and openly accessible foundational LLM with parameters ranging from 7 billion to 65 billion^14^. Instruction-following models fine-tuned from LLaMA such as Alpaca^20^ and Vicuna^21^, exhibit performance on par with GPT-3.5. Within the medical context, several groups have directed their efforts toward fine-tuning LLaMA. Notable examples among these are ChatDoctor (trained on authentic patient-physician dialogues), HuaTuo (fine-tuned with a Chinese medical knowledge graph), and PMC-LLaMA (fine-tuned on biomedical academic papers)^22–24^. These LLaMA-based models focused on medical question answering, yielding encouraging outcomes.

In this study, we demonstrated superior performance of the fine-tuned LLaMA in the text classification task of DRG prediction. Previous studies have underscored the effectiveness of employing diverse machine learning algorithms and deep neural networks for DRG prediction within healthcare systems outside the United States^10,11^. These studies focused on using structured data as input variables instead of clinical text. More recently, CAML model exhibited superior ability to predict DRGs^13^. CAML model, exclusively utilizing clinical notes, surpassed the performance of a Long Short-Term Memory (LSTM) model using structured clinical variables^13^. When compared with ClinicalBERT, CAML provided improved F1 scores but lower AUC^13,15^. We observed that DRG-LLaMA outperformed prior leading models of ClinicalBERT and CAML.

ClinicalBERT and CAML already stand as robust baselines, with the added benefit of much faster training times (supplement Table 1). While BERT-based models have a maximum input length of 512 tokens, CAML has the flexibility to handle longer context^13,19^. We also observed that the performance of DRG-LLaMA enhanced with the utilization of larger models and longer input context length. Interestingly, a recent study revealed that the optimal performance of LLMs is attained when pertinent information is positioned at either the beginning or the end of the input context, with a decline as the input context expands^25^. In our constrained experiments conducted with a maximum input token limit up to 1024, we have yet to encounter this limitation. In our study, the performance of both the baseline models and DRG-LLaMA surpassed the outcomes reported in prior research^13^. Beyond the substantially larger training dataset employed in MIMIC-IV compared to MIMIC-III (236,192 vs. 17,815), it is plausible that this enhanced performance is predominantly linked to our strategic input data selection.

The study by Liu et al.^13^ included only clinical notes charted up to 48 hours post-admission or 48 hours after ICU admission. In the MIMIC-III database, a large portion of records during this time window comprises nursing and radiology notes, potentially lacking the pivotal admission History of Present Illness (HPI) notes. In contrast, our methodology entailed the utilization of discharge summaries as the input data source. Discharge summary is a comprehensive clinical narrative encapsulating pivotal events, diagnostics, and treatments during hospitalization. To accommodate the input token limitations of LLaMA, we exclusively focused on the “brief hospital course” section of the summary, intentionally excluding other segments such as physical examinations, radiology, laboratory, and medication list. Additionally, to enhance data consistency, we formulated an algorithm aimed at addressing discrepancies in DRG nomenclature and assignments across different years.

In the context of the DRG system, a DRG code comprises a base DRG and a CC/MCC status. The base DRG represents the principal diagnosis (for medical cases) or procedures (for surgical cases) leading to the patient’s admission. Meanwhile, CC/MCC categorizations gauge the severity of the patient’s condition. In the 34.0 version of the MS-DRG system, there are 154 three-way split DRGs, 44 two-way split DRGs with MCC/CC and no CC, 65 two-way split DRGs with MCC and CC/no CC, and 77 base DRGs with no splits (examples in Supplementary Note 1)^8^. We experimented to resemble this structure through a two-label DRG prediction strategy. Surprisingly, the top-1 accuracy for CC/MCC stands at 67.5%, similar to 67.8% of the base DRG despite the considerably smaller label count (5 labels in CC/MCC vs. 340 labels in base DRG). These unexpected results likely stem from the noisy nature of CC/MCC assignment. For instance, the DRG code “pulmonary edema and respiratory failure” does not have a CC/MCC split. Therefore, a hospital stay with this DRG code may truly contain MCC, but the MCC would not be labeled as positive in the training set. To address this challenge, we formulated rules in both the DRGs dissection phase (extracting base DRGs and CC/MCC from DRGs) and the inference phase (deriving DRGs based on base DRGs and CC/MCC). These rules cater to various split scenarios, thus improving accuracy. Implementing such rules has culminated in a final DRG prediction accuracy close to single-label prediction (51.5% vs. 52.0%).

Our error analysis also revealed intriguing observations. While certain vulnerabilities (e.g., erroneous CC/MCC classification and inadequate clinical concept extraction) present opportunities that theoretically can be addressed through employment of larger LLM and more data, other challenges likely stem from inherent limitations within our training data setup. For instance, in Case 2 in Table 4, despite the discharge summary providing a more comprehensive discussion on gastrointestinal bleeding compared to acute renal failure, the latter was deemed the correct base DRG. This selection is guided by the DRG assignment rule^8^, a factor extending beyond the scope of what is directly evident within the discharge summary.

Our study has several limitations. 1) We were limited by the constraints of the MIMIC-IV dataset and could only use discharge summaries as input data, which are only available after the patient is discharged from the hospital. However, an effective alternative for predicting early DRGs would be to utilize HPI notes and/or Emergency Department (ED) notes. This approach has the potential to significantly impact hospital operations. The “assessment and plan” in HPI notes are similar in structure to the “brief hospital course” in discharge summaries. Thus, LLMs might find it easier to extract information related to the principal diagnosis from these notes, given their earlier time stamp in the hospitalization process. 2) We were also restricted by computational resource limitations, so we could only experiment with the LLaMA model up to a parameter size of 13 billion. Unfortunately, we couldn’t perform an extensive hyperparameter search. The largest LLaMA models have over 65 billion parameters.

The results presented in this study highlight the potential of adapting LLMs for medical purposes, particularly in predicting DRGs. Future research should involve collaborating with healthcare systems and utilizing admission notes to enable early DRG prediction. Additionally, our findings suggest that experiments utilizing the latest LLMs, including the recently launched 70-billion-parameter LLaMA-2 model with a maximum context length of 4096 tokens^26^, should be considered. Finally, a crucial area for exploration concerns the practical implications of such DRG prediction, particularly when integrated into existing hospital coding workflows.

## Methods

### Dataset and preprocessing

We conducted a study using the publicly available MIMIC-IV dataset, which comprises 431,231 unique hospital admissions from 299,712 patients admitted to an ICU or the ED of the Beth Israel Deaconess Medical Center in Boston, Massachusetts^27^. The dataset covers the period from 2008 to 2019. We used regular expressions to extract the “brief hospital course” section from the discharge summary as input text. We then filtered the discharge summaries that were of low quality, identified by either duplicated content or containing less than 40 words.

Our focus was on hospitalizations with MS-DRGs. We consolidated all MS-DRG codes to version 34.0, published in 2016 (detailed in the subsequent section)^8^. This version comprises a total of 757 DRG codes, with 738 being represented in our dataset. We allocated 90% of the data to the training set and the remaining 10% to the testing set, stratified by DRG codes.

### Process to address different DRG versions

Centers for Medicare & Medicaid Service adjusts MS-DRG regulations annually, resulting in varying DRG assignments for identical conditions over time within the MIMIC-IV dataset^28^. To address this discrepancy, we designed an algorithm based on clinical knowledge to harmonize MS-DRG codes across different time points to a unified version—specifically, MS-DRG version 34.0^8^. The process include:

1. Standardize use of abbreviations and capitalization within DRGs. For example, we replaced all “W/O” to “WITHOUT”, “CATH” to “CATHETERIZATION” and “PROC” to “PROCEDURES”.
2. Using a fuzzy string match algorithm (TheFuzz: https://github.com/seatgeek/thefuzz) to find those DRGs not matching to any MS-DRG v.34 codes.
3. An internal medicine physician manually reviewed all DRG codes from step 2, and assigned these codes to the most appropriate MS-DRG v.34.0 codes if applicable. Subsequently, a domain expert specializing in inpatient Clinical Documentation Integrity (CDI) assessed the conversion table and independently verified the accuracy of the code assignments.
4. Of note, after above steps there are several historical DRGs left without appropriate DRG v.34 codes assignment. For example, “URINARY STONES W MCC” and “NASAL TRAUMA AND DEFORMITY WITH CC”. These hospitalizations were excluded from the cohort.
5. Lastly, we filtered out rare DRGs with less than 2 occurrences in our cohort.

### Model development

**Fig. 3:**
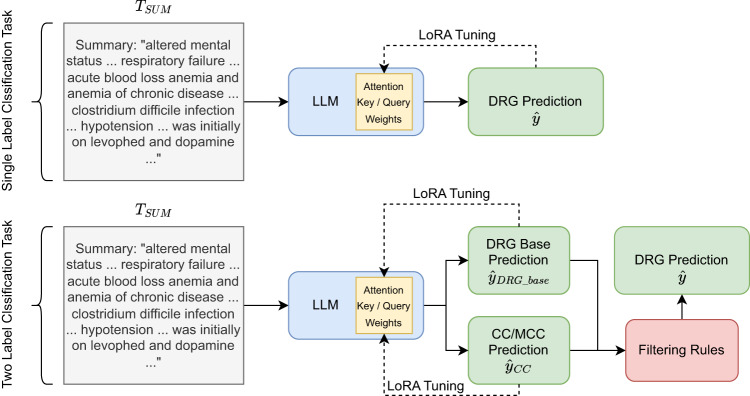
An illustration of both approaches we tested. Single Label Prediction–which directly predicts the DRG code from the text–as well as Two Label Prediction–which breaks down the classification task into 2 tasks. The two predictions are then combined using filtering rules (discovered from data for each DRG) at inference time for the final DRG prediction. LoRA training is used to train the LLM due to computational constraints.

We performed fine-tuning of the LLaMA model using discharge summaries and DRG codes within the context of a classification task. Our approach includes two distinctive strategies (also shown in Fig. 3).

#### Single label approach

In this approach, the model generates a single-label multi-class prediction for the DRG code from a training set of natural text discharge summaries *T*_*SUM*_ and labels containing $({T}_{SUM,i},{y}_{i})\in {{{\bf{{{{\mathcal{D}}}}}}}}$. We omit the index notation *i* for the rest of the descriptions without loss of generality. First, let us tokenize *T*_*SUM*_ based on the LLaMA Tokenizer into ***K*** = *tokenize*(*T*_*SUM*_). ***K*** is a list of indices that index into learnable embedding weights. Let *LLM*() be a function that outputs the embedding for each token after running the transformer model. Finally, the raw logits are calculated as

1 $$\hat{{{{\boldsymbol{y}}}}}=LLM{({{{\boldsymbol{K}}}})}_{-1}$$

where we use the last token embedding of *LLM*(***K***) as the predicted raw logit score of each DRG code $\hat{{{{\boldsymbol{y}}}}}\in {{\mathbb{R}}}^{738}$. Note that this logit score is the raw, unnormalized output of the last layer of the LLM. Before applying the activation function like the softmax function, which converts these scores to probabilities, the values produced by the network are referred to as logits.

The conventional categorical cross-entropy loss function for multi-class classification is used. i.e., a classic multi-class problem with loss: the target DRG *y* is an integer between 0 and 737 (note that we use an integer representing a specific DRG code for simplicity).

2 $$\ell (\hat{{{{\boldsymbol{y}}}}},y)=-\log \frac{\exp ({\hat{{{{\boldsymbol{y}}}}}}_{y})}{\mathop{\sum }\nolimits_{c = 1}^{C}\exp ({\hat{{{{\boldsymbol{y}}}}}}_{c})}$$

Where *y* ∈ {0, 1, …, 737} is the target DRG, and ${\hat{{{{\boldsymbol{y}}}}}}_{c}$ is the *c*^*th*^ index of $\hat{{{{\boldsymbol{y}}}}}$.

#### Two-label approach

In contrast, the two-label approach entails the model initially predicting the base DRG and the CC/MCC status as two separate classification tasks. Subsequently, a mapping rule (detailed in the subsequent section) is applied to derive DRG code. This approach entailed a loss function configured as the cross-entropy loss of the base DRG, plus half of the cross-entropy loss of the CC/MCC status.

More formally,

3 $$\ell (\hat{{{{\boldsymbol{y}}}}},y)={\ell }_{DRG\_base}({\hat{{{{\boldsymbol{y}}}}}}_{DRG{{{\_}}}base},{y}_{DRG{{{\_}}}base})+\lambda {\ell }_{CC}({\hat{{{{\boldsymbol{y}}}}}}_{CC},{y}_{CC})$$

Where ${\ell }_{DRG\_base}({\hat{{{{\boldsymbol{y}}}}}}_{DRG{{{\_}}}base},{y}_{DRG{{{\_}}}base})$ and ${\ell }_{CC}({\hat{{{{\boldsymbol{y}}}}}}_{CC},{y}_{CC})$ are also categorical cross entropy losses. We chose $\lambda =\frac{1}{2}$ for our work. As shown in Table 3, *y*_*DRG*_*base*_ ∈ {0, 1, …, 339} and *y*_*CC*_ ∈ {0, …, 4}, representing the categories of ["without CC/MCC”, “with CC”, “with MCC”, “without MCC”, and “not applicable”] respectively.

To enable ease of implementation, we used an output logit dimension of $\hat{{{{\boldsymbol{y}}}}}\in {{\mathbb{R}}}^{340+5}$ and indexed the first 340 dimensions for ${\hat{{{{\boldsymbol{y}}}}}}_{DRG\_base}={\hat{{{{\boldsymbol{y}}}}}}_{0,\ldots ,339}$ and indexed the last 5 dimensions for ${\hat{{{{\boldsymbol{y}}}}}}_{CC}={\hat{{{{\boldsymbol{y}}}}}}_{340,\ldots ,344}$. At inference time, we take the base DRG and CC/MCC predictions as the argmax of their respective logits.

4 $${\hat{y}}_{DRG\_base}=argma{x}_{{\hat{y}}_{DRG\_base}}({\hat{{{{\boldsymbol{y}}}}}}_{DRG\_base})$$

5 $${\hat{y}}_{CC}=argma{x}_{{\hat{y}}_{CC}}({\hat{{{{\boldsymbol{y}}}}}}_{CC})$$

Subsequently, we apply the mapping rule, as detailed below, to derive the final DRG prediction from base DRG and CC/MCC labels.

#### Process to dissect and derive DRGs to/from base DRGs and CC/MCC

We first used regular expression to obtain principal diagnosis/procedures in MS-DRG v.34.0, by extracting strings prior to the description of CC/MCC. For example, in DRG 11 of “tacheostomy for face mouth and neck diagnoses with mcc”, the principal diagnosis is “tacheostomy for face mouth and neck diagnoses”. After this step, 340 principal diagnosis/procedures are identified as base DRGs.

We assigned CC/MCC status to one of the five labels: “without CC/MCC,” “with CC,” “with MCC,” “without MCC,” and “not applicable”. Of note, an important detail is that if a DRG code does not explicitly describe CC/MCC status, we will assign a label of “not applicable”. Such an example is DRG 69 “transient ischemia”. We realize such classification might bring in noisy signals for models to learn (as a patient with “transient ischemia” can indeed has CC/MCC), but found it better than assigning to an alternative label such as “without CC/MCC” which would be more erroneous.

When inferencing DRGs from base DRGs and CC/MCC, we developed a rule based on logic and clinical knowledge. First, we evaluate whether predicted principal diagnosis/procedure matches target base DRG. Second, if the predicted CC/MCC label is in the CC/MCC set of the target base DRG, we make comparison directly. Third, for those predicted CC/MCC labels not in the CC/MCC set of the target base DRG, we apply a mapping procedure based on different MS-DRG splits as listed in Supplementary Note 1. For example, if a MS-DRG code has no split, such as DRG 69 “transient ischemia”, then any CC/MCC predictions can be mapped to the correct DRG (as long as the base DRG matches). Another example would be MS-DRG 56 and 57, where there are two splits of CC/MCC status ("with MCC” and “without MCC”). In this case we will map predictions of “without CC/MCC”, “with CC” and “not applicable” all to the label of “without MCC” for final inference.

#### Addressing Computational Constraints via LoRA Training

Given the constraints of available computational resources, an extensive hyperparameter search was not viable. Instead, our focus encompassed exploring the performance across diverse model sizes and token lengths. We used LoRA during training, which involves freezing the pre-trained model weights and incorporating trainable rank decomposition matrices into each layer of the transformer architecture^29^. Lora training of the attention mechanism is shown in Fig. 3.

As a quick summary, let us assume that we have original weight matrix ${{{{\boldsymbol{W}}}}}_{{{{\boldsymbol{0}}}}}\in {{\mathbb{R}}}^{d\times k}$. LoRA works by adding a low-rank matrix to the original weight matrix: Δ***W*** + ***W***_**0**_, Δ***W*** = ***BA*** where ${{{\boldsymbol{B}}}}\in {{\mathbb{R}}}^{d\times r}$ and ${{{\boldsymbol{A}}}}\in {{\mathbb{R}}}^{r\times k}$. Note that one should choose $r\ll \min (d,k)$ and only adapt the attention weights to ensure constraints on the dimensionality of the new weights and preserve original model performance. Training is only performed on this Δ***W***, and original model weights are kept the same. We also only tune the weights of the attention mechanism for further cost savings while preserving performance.

#### Training Details

Model training adopted standard Huggingface training framework and the sequence classification module^30^. Since LLaMA is a decoder-only (causal) model, we follow the traditional approach of using the embedding of the last token to do the classification, as other causal models (e.g. GPT-2^31^) do. Logits score of each DRG label was calculated from this linear output layer, and probabilities of DRGs could be derived using a softmax function.

We referenced the training protocol of Alpaca-Lora^32^. The model was quantized to 8-bit integer using bitsandbytes library^33^. Our model was trained using cross-entropy loss with the AdamW optimizer (learning rate = 2 × 10^−5^ and weight decay = 0.01) for 3 epochs on all training data and batch size of 4. Lora parameters were configured with r set to 8, an alpha value of 16, and a dropout rate of 0.05. All attention blocks were included in the Lora target modules. The training regimen for all DRG-LLaMA models were executed on a singular Nvidia RTX A6000 GPU with 48GB of graphics memory.

### Baseline models

As baseline models for benchmarking, we selected CAML^12,13^ and ClinicalBERT^15^. CAML is an adjusted convolutional neural network (CNN). In CAML, clinical notes are tokenized and embedded with pre-trained word embeddings to form input representations. Subsequently, inputs are passed on to a neural network with one-dimensional convolutions that pool CNN features using the attention mechanism. In line with the approach detailed in^13^, our training of CAML included early stop when there was no improvement in micro-averaged F1 score for 10 consecutive epochs, with a maximum epochs of 50. All default hyperparameters were kept, except for max_seq_length which was set to 512.

ClinicalBERT was built upon BioBERT, a domain-specific BERT model pre-trained on PubMed abstracts and full-text articles from PubMed Central^16^. ClinicalBERT performed further pre-training of BioBERT using 2 million clinical notes from MIMIC-III^34^. In our fine-tuning process of ClinicalBERT, we conducted three training epochs, same as DRG-LLaMA. We set a learning rate of 2 × 10^−5^ and a batch size of 16, consistent with previous recommended practice for classification-oriented fine-tuning of BERT^19,35^.

### Statistical analysis

We used the implementation from^13^ to calculate AUC and F1-score in both macro- and micro- approach for predictive models. We also reported accuracy of DRG prediction at top one, five and ten results. Standard deviations were calculated using a bootstrapping procedure with 30 iterations. For each bootstrap iteration, we randomly resampled the whole sample size from the testing set with replacement. Smoothing spline fit in Fig. 2a was performed using npreg package in R with generalized cross-validation method and default parameters^36^.

### Ethical concerns

MIMIC-IV is a free EHR dataset that is deidentified according to the Health Insurance Portability and Accountability Act (HIPAA) Safe Harbor provision^27^

Since we primarily used open source models such as LLaMA and ClinicalBERT from Huggingface, an open source repository of machine learning models^30^ as well as CAML from github, and trained it on MIMIC, privacy risks are quite low. However, this risk should not be counted out when working with LLMs, and it is possible that LLaMA and ClinicalBERT may be trained on sensitive data in their respective pretrainining stages.

### Reporting summary

Further information on research design is available in the Nature Research Reporting Summary linked to this article.

### Supplementary information

SUPPLEMENTAL MATERIAL

Reporting Summary

## Data Availability

Access to MIMIC-IV can be requested at https://physionet.org/content/mimiciv/, which requires a signed safe usage agreement.

## Code availability

Scripts for this work were written in Python. They are available with accompanied documentation at https://github.com/hanyin88/drg-llama.
