# Supplementary material for: DRG-LLaMA : tuning LLaMA model to predict diagnosis-related group for hospitalized patients
Source: NPJ Digit Med. 2024 Jan 22;7:16. doi: 10.1038/s41746-023-00989-3 (PMC10803802; doi:10.1038/s41746-023-00989-3)
Supplement: Supplementary file 1 — SUPPLEMENTAL MATERIAL [file 41746_2023_989_MOESM1_ESM.pdf]

Supplement Materials

1 Supplementary Note 1: Examples of DRGs split in MS-DRG version 34.0

1. Three-way split DRGs:
    - DRG 11: tracheostomy for face mouth and neck diagnoses with MCC
    - DRG 12: tracheostomy for face mouth and neck diagnoses with CC
    - DRG 13: tracheostomy for face mouth and neck diagnoses without CC/MCC
2. Two-way spit DRGs with MCC/CC and no CC:
    - DRG 52: spinal disorders and injuries with CC/MCC
    - DRG 53: spinal disorders and injuries without CC/MCC
3. Two-way split DRGs with MCC and CC/no CC:
    - DRG 56: degenerative nervous system disorders with MCC
    - DRG 57: degenerative nervous system disorders without MCC
4. Base DRGs with no splits:
    - DRG 69: transient ischemia

2 Supplementary Note 2: Further discussion on the motivation for DRG prediction

Pragmatically, the approach of our work (of using discharge summary as input data) is restrained by the availability of public clinical notes and DRG labels. Notably, the MIMIC-IV database, the largest publicly accessible resource in our field, exclusively offers discharge summaries. It is pertinent to underscore that, even with our current model configuration, which predicts DRGs based on discharge summaries, substantial value likely exists. In many healthcare systems, DRGs are routinely assigned by Clinical Documentation Integrity (CDI) specialists several days post-patient discharge. Consequently, our model can still furnish timely DRG information in many instances, thereby contributing to the informed and streamlined management of hospital operations.

In our manuscript, we elucidated the structural and compositional parallels between discharge summaries and admission History of Present Illness (HPI) notes. Therefore, it would be reasonable to hypothesize DRG-LLaMA could predict DRGs effectively when using HPI notes as input data. Furthermore, encouraged by the promising outcomes of our proof-of-concept work in this manuscript, we have submitted a research proposal to refine our model further using admission HPI notes accessible within a large healthcare system, such as the Mayo Clinic. The earlier prediction of DRGs, made possible by this approach, holds significant potential for

enhancing daily hospital operations. For instance, within hospital medicine practices, there's a strong interest in identifying patients associated with the shorter geometric length of stay (a metric bundled to DRG code). This aids in prioritizing early discharges opportunities at the start of the day. Similarly, an early grasp of the case mix index (another DRG-bundled metric) can guide resource allocation decisions, such as adjusting the nurse-to-patient ratio based on the level of severity.

3 Supplementary Discussion

3.1 Distribution of training cases per DRG

The distribution of training cases per DRG is quite imbalanced with a long tail (Supplementary Figure 1). The median training cases per DRG is 124.5, and there are only 12 DRG codes with a training cases exceeding 2000.

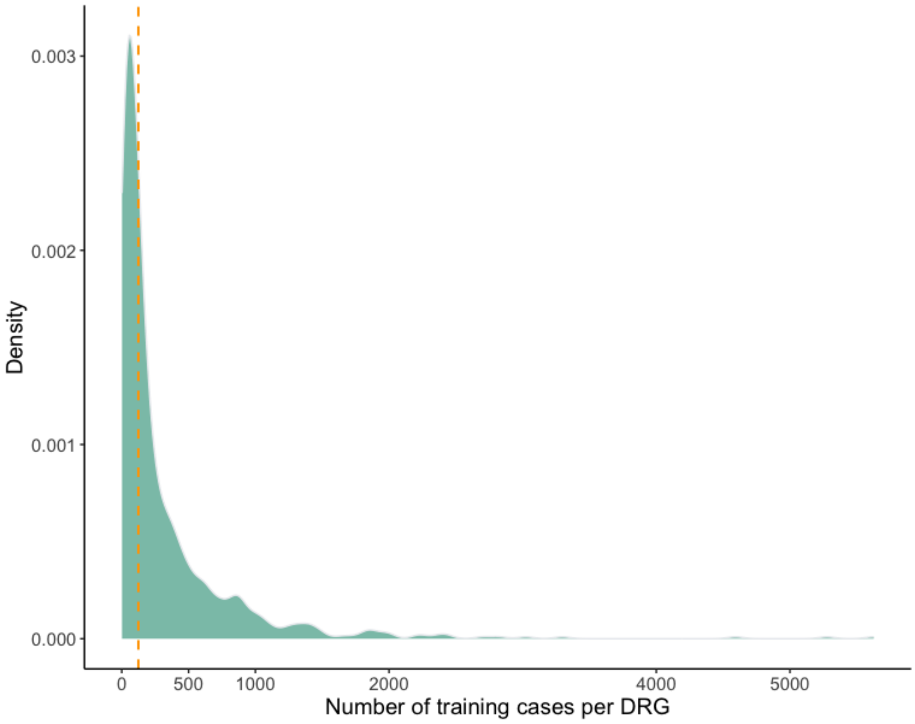

Supplementary Figure 1: Density plot showing distribution of training cases per DRG. Density plots are conceptually equivalent to histograms, but use probability density to display where values are concentrated. The dashed line represent the median number of training cases.

3.2 Computational cost

The training and evaluation times for all models are presented in Supplementary Table 1. Notably, ClinicalBERT and CAML exhibited considerably shorter duration to train in comparison to `DRG-LLaMA` . As the maximal input token size doubled, the training time for `DRG-LLaMA` increased by approximately 90%. Moreover, when `DRG-LLaMA` model size expanded from 7 billion to 13 billion parameters, the training time showed an increment of roughly 70%.

Supplementary Table 1: Computational costs of all models

Model	Max input token size	Training time (hours)	Evaluation steps per second
DRG-LLaMA -7B	340	104	2.39
	512	151	1.70
	1024	285	0.84
DRG-LLaMA -13B	340	177	1.54
	512	258	1.08
	1024	498	0.52
ClinicalBERT	512	5	7.26
CAML	512	0.8	98.53

All training and evaluation were performed on one 48GB Nvidia RTX A6000 GPU. Batch sizes for DRG-LLaMA , ClinicalBERT and CAML were 4, 16 and 32, respectively.

3.3 Analysis of DRGs with best and worst prediction performance

The nine DRGs with a top-1 prediction accuracy of 100% by DRG-LLaMA are detailed in Supplementary Table 2. Majority of these DRGs (8 out of 9) fall within the category of surgical DRGs, representing infrequent yet highly specialized procedures. Interestingly, the median number of training cases for this particular set of DRGs is low at 23. Supplementary Table 3 and Table 4, on the other hand, outline medical and surgical DRGs with a top-5 prediction accuracy of 0%, respectively. Within this cohort, the median number of training samples is 17.5 for medical DRGs and 16 for surgical DRGs. Among these challenging-to-predict DRGs, many are clinically and semantically difficult to differentiate from alternative options. For instance, consider the distinction between DRG 671, “Urethral Procedures With CC/MCC,” and DRG 668, “Transurethral Procedures With MCC.”

Supplementary Table 2: DRGs with top-1 prediction accuracy of 100% by DRG-LLaMA

MS-DRG Code	Description	Medical or Surgical	No. of training cases
777	Ectopic Pregnancy	Medical	59
801	Splenectomy Without CC/MCC	Surgical	23
8	Simultaneous Pancreas and Kidney Transplant	Surgical	21
10	Pancreas Transplant	Surgical	19
799	Splenectomy With MCC	Surgical	23
652	Kidney Transplant	Surgical	380
116	Intraocular Procedures With CC/MCC	Surgical	17
16	Autologous Bone Marrow Transplant With CC/MCC	Surgical	155
114	Orbital Procedures Without CC/MCC	Surgical	31

Results from DRG-LLaMA -7B with an input token window of 512.

Supplementary Table 3: Medical DRGs with top-5 prediction accuracy of 0% by DRG−LLaMA

MS-DRG Code	Description	No. of training cases
53	Spinal Disorders and Injuries Without CC/MCC	17
63	Acute Ischemic Stroke With Use of Thrombolytic Agent Without CC/MCC	14
67	Nonspecific CVA and Precerebral Occlusion Without Infarction With MCC	17
77	Hypertensive Encephalopathy With MCC	21
79	Hypertensive Encephalopathy Without CC/MCC	6
80	Nontraumatic Stupor and Coma With MCC	33
81	Nontraumatic Stupor and Coma Without MCC	78
88	Concussion With MCC	12
89	Concussion With CC	25
90	Concussion Without CC/MCC	31
122	Acute Major Eye Infections Without CC/MCC	13
124	Other Disorders of the Eye With MCC	35
150	Epistaxis With MCC	32
182	Respiratory Neoplasms Without CC/MCC	19
284	Acute Myocardial Infarction Expired With CC	20
285	Acute Myocardial Infarction Expired Without CC/MCC	10
290	Acute and Subacute Endocarditis Without CC/MCC	8
297	Cardiac Arrest Unexplained With CC	10
383	Uncomplicated Peptic Ulcer With MCC	17
533	Fractures of Femur With MCC	11
537	Sprains Strains and Dislocations of Hip Pelvis and Thigh With CC/MCC	18
538	Sprains Strains and Dislocations of Hip Pelvis and Thigh Without CC/MCC	7
548	Septic Arthritis With MCC	12
550	Septic Arthritis Without CC/MCC	12
594	Skin Ulcers Without CC/MCC	15
598	Malignant Breast Disorders With CC	43
688	Kidney and Urinary Tract Neoplasms Without CC/MCC	6
695	Kidney and Urinary Tract Signs and Symptoms With MCC	25
722	Malignancy Male Reproductive System With MCC	19
725	Benign Prostatic Hypertrophy With MCC	19
730	Other Male Reproductive System Diagnoses Without CC/MCC	6
756	Malignancy Female Reproductive System Without CC/MCC	8
757	Infections Female Reproductive System With MCC	23
836	Acute Leukemia Without Major O.R. Procedure Without CC/MCC	26
845	Other Myeloproliferative Disorders or Poorly Differentiated Neoplastic Diagnoses Without CC/MCC	16
886	Behavioral and Developmental Disorders	30
887	Other Mental Disorder Diagnoses	30
913	Traumatic Injury With MCC	21

Results from DRG−LLaMA -7B with an input token window of 512.

Supplementary Table 4: Surgical DRGs with top-5 prediction accuracy of 0% by DRG-LLaMA

MS-DRG Code	Description	No. of training cases
13	Tracheostomy for Face Mouth and Neck Diagnoses Without CC/MCC	13
115	Extraocular Procedures Except Orbit	24
117	Intraocular Procedures Without CC/MCC	5
130	Major Head and Neck Procedures Without CC/MCC	13
135	Sinus and Mastoid Procedures With CC/MCC	8
138	Mouth Procedures Without CC/MCC	26
231	Coronary Bypass With PTCA With MCC	14
232	Coronary Bypass With PTCA Without MCC	11
241	Amputation for Circulatory System Disorders Except Upper Limb and Toe Without CC/MCC	23
257	Upper Limb and Toe Amputation for Circulatory System Disorders Without CC/MCC	22
258	Cardiac Pacemaker Device Replacement With MCC	16
263	Vein Ligation and Stripping	16
332	Rectal Resection With MCC	26
341	Appendectomy Without Complicated Principal Diagnosis With MCC	16
344	Minor Small and Large Bowel Procedures With MCC	50
347	Anal and Stomal Procedures With MCC	30
410	Biliary Tract Procedures Except Only Cholecyst With or Without C.D.E. Without CC/MCC	35
411	Cholecystectomy With C.D.E. With MCC	7
412	Cholecystectomy With C.D.E. With CC	19
413	Cholecystectomy With C.D.E. Without CC/MCC	14
422	Hepatobiliary Diagnostic Procedures Without CC/MCC	18
425	Other Hepatobiliary or Pancreas O.R. Procedures Without CC/MCC	14
458	Spinal Fusion Except Cervical With Spinal Curvature or Malignancy or Infection or Extensive Fusions Without CC/MCC	9
487	Knee Procedures With PDX of Infection Without CC/MCC	25
507	Major Shoulder or Elbow Joint Procedures With CC/MCC	17
518	Back and Neck Procedures Except Spinal Fusion With MCC or Disc Device or Neurostimulator	35
576	Skin Graft Except for Skin Ulcer or Cellulitis With MCC	14
624	Skin Grafts and Wound Debridement for Endocrine Nutritional and Metabolic Disorders Without CC/MCC	13
630	Other Endocrine Nutritional and Metabolic O.R. Procedures Without CC/MCC	22
662	Minor Bladder Procedures With MCC	7
666	Prostatectomy With CC	10
671	Urethral Procedures With CC/MCC	14
711	Testes Procedures With CC/MCC	12
715	Other Male Reproductive System O.R. Procedures for Malignancy With CC/MCC	9
716	Other Male Reproductive System O.R. Procedures for Malignancy Without CC/MCC	8
802	Other O.R. Procedures of the Blood and Blood Forming Organs With MCC	31
829	Myeloproliferative Disorders or Poorly Differentiated Neoplasms With Other O.R. Procedure With CC/MCC	68
830	Myeloproliferative Disorders or Poorly Differentiated Neoplasms With Other O.R. Procedure Without CC/MCC	17
855	Infectious and Parasitic Diseases With O.R. Procedure Without CC/MCC	13
901	Wound Debridements for Injuries With MCC	23
903	Wound Debridements for Injuries Without CC/MCC	32
928	Full Thickness Burn With Skin Graft or Inhalation Injury With CC/MCC	6
939	O.R. Procedures With Diagnoses of Other Contact With Health Services With MCC	29
959	Other O.R. Procedures for Multiple Significant Trauma Without CC/MCC	55
970	HIV With Extensive O.R. Procedure Without MCC	10

Results from DRG-LLaMA -7B with an input token window of 512.
